# Preclinical evidences toward the use of triterpenoid CDDO-Me for solid cancer prevention and treatment

**DOI:** 10.1186/1476-4598-13-30

**Published:** 2014-02-20

**Authors:** Yan-Yang Wang, Hong Zhe, Ren Zhao

**Affiliations:** 1 Department of Radiation Oncology, General Hospital of Ningxia Medical University, No.804 Shengli Str, Yinchuan, Ningxia, China

**Keywords:** Triterpenoid, CDDO-Me, Solid cancer, Prevention, Treatment, Mechanisms

## Abstract

Solid cancer remains a major cause of death in the world. As limited treatment options are currently available to patients with solid cancer, novel preventive control and effective therapeutic approaches are considered to be reasonable and decisive measures to combat this disease. The plant-derived triterpenoids, commonly used for medicinal purposes in many Asian countries, poses various pharmacological properties. A large number of triterpenoids exhibit cytotoxicity against a variety of cancer cells, and cancer preventive, as well as anticancer efficacy in preclinical animal models. To improve antitumor activity, some synthetic triterpenoid derivatives have been synthesized, including cyano-3,12-dioxooleana-1,9(11)- dien-28-oic (CDDO), its methyl ester (CDDO-Me), and imidazolide (CDDO-Im) derivatives. In this review, we will critically examine the current preclinical evidences of cancer preventive and therapeutic activity about one of the synthetic triterpenoids, CDDO-Me. Both in vitro and in vivo effects of this agent and related molecular mechanisms are presented.

## Introduction

It has now become evident that cancer development underlies a collection of multiple genetic abnormalities through a multistep, mutagenic process [1,2]. The complexity of the genetic lesions and the variation of the derivative phenotypic changes of cancer cells indicate that multifunctional drugs capable of affecting cancer cells at various levels must be used for the treatment of these diseases [3,4]. With this background, agents like triterpenoids that can alter multiple dysregulated cellular pathways may have a significant potential for solid cancer prevention and treatment [5,6]. In this review, we will critically examine the current preclinical evidences of cancer preventive and therapeutic activity about one of the synthetic triterpenoids, C-28 methyl ester of 2-cyano-3,12-dioxoolean-1,9-dien-28-oic acid (CDDO-Me). Both in vitro and in vivo effects of this agent and related molecular mechanisms are presented.

### Introduction of CDDO-Me

Triterpenoids are metabolites of isopentenyl pyrophosphate oligomers and represent the largest group of phytochemicals. It has been estimated that more than 20,000 triterpenoids exist in nature [7]. Triterpenoids are used medicinally in many Asian countries for their anti-inflammatory, analgesic, antipyretic, hepatoprotective, cardiotonic, sedative and tonic effects [8,9]. An increasing number of triterpenoids have been reported to exhibit cytotoxicity against a variety of cancer cells without manifesting any toxicity in normal cells. They also demonstrate antitumor efficacy in preclinical animal models of cancer [4,10,11].

To improve antitumor activity, triterpenoid derivatives have been synthesized. In 1999, one such synthetic triterpenoid, 2-cyano-3,12-dioxoolean-1,9-dien-28-oic acid (CDDO), which has been found to be 100–500 fold more potent than any previous triterpenoids in suppressing inducible nitric oxide synthase and cyclooxygenase 2 (COX-2) – two inflammatory enzymes having important roles in the development of malignancy [12,13] , has been successfully developed [14]. In order to increase the potency of CDDO, various C-28 derivatives have been synthesized. CDDO-Me, one of the derivatives of CDDO, has shown the effects of preventing or treating cancer in a variety of studies [5]. The antiproliferative effect exerted by CDDO-Me in cancer cells seems to be more potent than that induced by either C-28 imidazolide of CDDO (CDDO-Im) or CDDO itself [15]. CDDO-Me is one of the most potent activators of the Nuclear factor (erythroid-derived 2)-like 2 (Nrf2) pathway. Biological response to CDDO-Me is dependent on dose. Low concentrations (nanomolar) of CDDO-Me target kelch-like ECH-associated protein 1 (Keap1) and activate the Nrf2/ antioxidant responsive element (ARE) cytoprotective and anti-inflammatory response. As the concentration of CDDO-Me increases, it targets Actin-related protein 3 (Arp3) and other components of the cytoskeleton to inhibit cell proliferation, whereas even higher concentrations (micromolar) of CDDO-Me can selectively induce apoptosis in cancer cells by targeting a number of key regulatory proteins and pathways that are frequently constitutively activated or overexpressed in cancer cells [5].

### In vivo cancer preventive effects of CDDO-Me

Anticarcinogenic activities of CDDO-Me have been established in both chemical carcinogenesis models and transgenic mouse models of various cancers (Table 1).

**Table 1:** ***In vivo *** **solid cancer preventive effects of CDDO-Me**

**Effects**	**Mechanisms**	**Dose/duration**	**Route**	**Reference**
Decreased the average tumor burden of vinyl carbamate-induced andenocarcinoma in female A/J mice	Suppression of the ability of IFN-γ to induce de novo formation of NO synthase; Induction of HO-1; Suppression of phosphorylation of STAT3 as well as induction of apoptosis	60 mg/kg, 2 weeks	Diet	[16]
Prevented the formation of ER- negative mammary tumors in the mouse mammary tumor virus-neu transgenic model	Inhibited constitutive STAT3 phosphorylation and the degradation of IKBα	60 mg/kg, 45 weeks	Diet	[17]
Increased survival of the KPC mice by 3 to 4 weeks	Interacted with both STAT3 and IKK to decrease constitutive IL-6 secretion, inhibited constitutive STAT3 phosphorylation, and blocked the degradation of IKBα when challenged with TNF-α	60 mg/kg diet or 15 mg/kg body weight. Beginning at 4 weeks of age until detect tumor	Diet	[18]
Delayed ER–negative mammary carcinogenesis in polyoma middle T Mice	Inhibited cyclin D1 and decreased phosphorylation of EGFR and STAT3	50 mg/kg, 4–8 weeks	Diet	[19]
Delayed tumor development in the BRCA1-mutated mice by an average of 5.2 weeks	Interacted with ErbB2, decreased constitutive phosphorylation of ErbB2, inhibited proliferation, and induced G0/G1 arrest	50 mg/kg. Beginning at 12 weeks of age until detect tumor	Diet	[20]
Inhibited the progression of preneoplastic lesions in the DLP and VP lobes to adenocarcinoma in TRAMP mice	Inhibited cell proliferation, reduced the density of blood vessels and promoted apoptosis in the prostatic tissue	10 μmol/kg. 20 weeks	Oral gavage	[21]
Inhibited the progression of the preneoplastic lesions to adenocarcinoma in the DLP and VP lobes of TRAMP mice	Inhibited the expression of prosurvival p-Akt and NF-κB in the prostate	7.5 mg/kg/day, 5 days/week. Early intervention initiated at five weeks of age for 20 weeks. Delayed administration started at 12 week of age for 12 weeks	Oral gavage	[22]
Inhibited the progression of preneoplastic lesions to adenocarcinoma of the prostate in TRAMP mice	Decreased TERT and its regulatory proteins in the prostate	15 μmol/kg/day, 5 days/week, 20 weeks	Oral gavage	[23]

Akt: protein kinase B1; BRCA1:breast cancer-associated gene 1; DLP: dorsolateral prostate; EGFR: epidermal growth factor receptor; ER: estrogen receptor; ErbB2: human epidermal growth factor receptor 2; HO-1: heme oxygenase-1; IKBα, inhibitor of nuclear factor-κBα; IKK:IKB kinase; IL-6: interleukin-6; IFN-γ: Interferon-γ; KPC; LSL-Kras^G12D/+^; LSL-Trp53^R127H/+^; Pdx-1-Cre; NF-κB: nuclear factor-κB; NO: nitric oxide; STAT3: transcription factor signal transducers and activators of transcription 3; TERT: telomerase reverse transcriptase TNF-α: tumor necrosis factor α; TRAMP: transgenic adenocarcinoma of the mouse prostate model; VP: ventral prostate.

CDDO-Me is a potent inhibitor of lung carcinogenesis in A/J mice. Liby et al. [16] looked at female A/J mice treated with the mutagenic carcinogen vinyl carbamate, which induces adenocarcinoma of the lung in all animals within 16 weeks. If mice were fed either the CDDO-Me or the CDDO ethyl amide (CDDO-Ea), beginning 1 week after dosing with carcinogen, the number, size, and severity of lung carcinomas were markedly reduced. The triterpenoids CDDO-Ea and CDDO-Me reduced the average tumor burden (ATB) in the lungs 86% to 92%, respectively, compared with the controls, and the rexinoid LG100268 reduced ATB by 50%.

Liby et al. [17] also investigated whether the CDDO-Me and the rexinoid LG100268 could prevent the formation of estrogen receptor (ER)-negative mammary tumors in the mouse mammary tumor virus-neu transgenic model. Mice were fed control diet, CDDO-Me, LG100268, or the combination. CDDO-Me and LG100268 significantly delayed the development of ER-negative tumors, with a 14- and 24-week delay, respectively, compared with the control group for the time required to reach 50% tumor incidence. The combination of CDDO-Me and LG100268 was significantly more potent than the individual drugs, as only one tumor was found in the combination group after 45 weeks on diet, at which time all control animals had tumors. The same group also found that CDDO-Me, LG100268, the combination of CDDO-Me and LG100268, and the combination of CDDO-Ea and LG100268, all significantly increased the survival of the LSL-Kras^G12D/+^; LSL-Trp53^R127H/+^; Pdx-1-Cre (KPC) mouse pancreatic cancer model by 3 to 4 weeks [18].

Tran et al. [19] tested the activity of CDDO-Me in a relevant model of ER–negative breast cancer, the polyoma-middle T (PyMT), in which the oncoprotein drives carcinogenesis. Mice were fed CDDO-Me, starting at 4 weeks of age. CDDO-Me significantly increased the age of mice at onset of first tumor by an average of 4.3 weeks and overall survival by 5.2 weeks. The drug also inhibited the infiltration of tumor-associated macrophages into mammary glands of PyMT mice at 12 weeks of age and reduced levels of the chemokines CXCL12 and CCL2 in primary PyMT mammary tumor cells. Treatment with this multifunctional drug also inhibited secretion of matrix metalloproteinase-9 in primary tumor cells from PyMT mice and decreased proliferation of these cells by inhibiting cyclin D1 and decreasing phosphorylation of epidermal growth factor receptor and signal transducer and activator of transcription 3 (STAT3).

In a mouse model in which deletion of the BRCA1 gene (breast cancer associated gene 1) is combined with a mutation in a single allele in the p53 tumor suppressor gene, CDDO-Me significantly delays tumor development [20]. Beginning at 12 weeks of age, Brca1^Co/Co^; MMTV-Cre;p53^+/-^ mice were fed powdered control diet or diet containing CDDO-Me. CDDO-Me significantly delayed tumor development in the BRCA1-mutated mice by an average of 5.2 weeks. The authors also observed that levels of ErbB2, pErbB2, and cyclin D1 increased in a time-dependent manner in the mammary glands in BRCA1-deficient mice, and CDDO-Me inhibited the constitutive phosphorylation of ErbB2 in tumor tissues from these mice. In BRCA1-deficient cell lines, the triterpenoids directly interacted with ErbB2, decreased constitutive phosphorylation of ErbB2, inhibited proliferation, and induced G0/G1 arrest. These results suggest that CDDO-Me has the potential to prevent BRCA1-mutated breast cancer.

Deeb et al. reported that administration of CDDO-Me to transgenic adenocarcinoma of the mouse prostate (TRAMP) mice resulted in inhibition of progression of preneoplastic lesions (low and high-grade prostate intraepithelial neoplasms, PINs) to adenocarcinoma of the prostate in more than 70% of the mice without noticeable toxicity [21,22]. Subsequently, the same group also found that treatment with CDDO-Me inhibited metastasis of cancer to lung, liver, kidney and pelvic lymph nodes. The response to CDDO-Me in TRAMP mice was associated with reduction in proteins that regulate production and phosphorylation of telomerase reverse transcriptase (TERT) [23].

### In vitro and in vivo anticancer effects of CDDO-me

In addition to its efficacy for prevention of cancer, CDDO-Me has been used to treat established tumors in experimental models (Table 2).

**Table 2:** ***In vitro *** **anti-cancer effects of CDDO-Me on various solid cancer cells**

**Cellular effects**	**Mechanisms**	**Concentration**	**Reference**
Inhibited proliferation and induced apoptosis by in LNCaP and PC-3 prostate cancer cell lines	Inhibited hTERT gene expression, hTERT telomerase activity and a number of proteins that regulate hTERT transcriptionally and posttranslationally	0.063-5 μM	[23]
Inhibited the growth of LNCaP, PC-3 and DU145 prostate cancer cells	Induced apoptosis through activation of caspases 3, 8 and 9, disruption of mitochondrial integrity, and inhibition of anti-apoptotic Bcl-2, Bcl-xL and XIAP. Induction of apoptosis was associated with the inhibition of pro-survival Akt, mTOR, NF-κB signaling proteins	1.25-20 μM	[26]
Inhibited the growth and induced apoptosis in PC-3 and C4-2 prostate cancer cells	Inhibition of p-Akt, mTOR, and NF-κB signaling proteins and their downstream targets such as p-Bad and p-Foxo3a [Akt]; p-S6K1, p-eIF-4E and p-4E-BP1 [mTOR]; and COX-2, VEGF and cyclin D1[NF-κB]	0.625-10 μM	[30]
Inhibited the growth of both K-ras mutated and wild-type K-ras pancreatic cancer cells	Inhibited of prosurvival p-Akt, NF-κB and mTOR signaling proteins and the downstream targets of Akt and mTOR, such as p-Foxo3a [Akt] and p-S6K1, p-eIF-4E and p-4E-BP1 [mTOR]	0.625- 5 μM	[32]
Inhibited the growth of colorectal cancer cells	Suppressed pro-survival Akt, NF-κB and mTOR signaling proteins and NF-κB-regulated anti-apoptotic Bcl-2, Bcl-xL, Bad and survivin	1.25-10 mM	[34]
Inhibited the growth of human ovarian cancer cell lines OVCAR-3, OVCAR-5 and SK-OV3 and ovarian endometrioid adenocarcinoma cell line MDAH-2774	Inhibition of Akt/ NF-κB /mTOR signaling pathway	0.625-10 μM	[36]
CDDO-Me caused the generation of ROS and pre-treatment with NAC prevented the generation of ROS in OVCAR-5 and MDA 2774 ovarian cancer cells	ROS played a pivotal role in the anti-proliferative and apoptosis-inducing activity of CDDO-Me in ovarian cancer cells	1.25–10 μM	[37]
Induced the apoptosis of glioblastoma [U87MG, U251MG] and neuroblastoma [SK-N-MC] cell lines	Inhibited the levels of anti-apoptotic and prosurvival p-Akt, NF-κB and Notch1 signaling molecules	2.5–10 μM	[38]
Reversed the resistant of paclitaxel-resistant ovarian cancer cell line OVCAR8_TR_ and cisplatin-resistant ovarian cancer cell line A2780cp70	Inhibited IL-6 secretion, STAT3 phosphorylation, STAT3 nuclear translocation	0.3 μM	[42]
Reversed the resistant of MDR osteosarcoma cell lines KHOS_R2_, U-2OS_TR_	Inhibited STAT3 phosphorylation, STAT3 nuclear translocation and induced apoptosis	0.1; 0.3 μM	[43]
Activated the extrinsic DR-mediated apoptotic pathway in human lung cancer cells	Induced a JNK-dependent up-regulation of DR5 expression, leading to activation of caspase-8 and induction of apoptosis	0.25-1 μM	[47]
Induced JNK-dependent DR5 expression in human lung cancer cells	Depleted intracellular GSH, resulting in ER stress. Subsequently, it activated JNK, leading to CHOP-dependent DR5 up-regulation and apoptosis	0.1-2 μM	[48]
Induced apoptosis in human lung cancer cells A549 and H157	Induced apoptosis through downregulation of c-FLIP Enhanced TRAIL-induced apoptosis	0.25;0.5;1 μM	[49]
Inhibited proliferation and induced apoptosis in MiaPaCa-2 and Panc-1 human pancreatic cancer cell lines	Inhibited hTERT gene expression, hTERT telomerase activity and a number of proteins that regulate hTERT expression and activity	1.25–5 μM	[54]
Induced apoptosis in MiaPaCa-2 and Panc-1 human pancreatic cancer cell lines	Generated ROS and inhibited the telomerase activity	1.25 μM	[55]
Inhibited MC38 colon carcinoma, Lewis lung carcinoma, and EL-4 thymoma mouse tumor lines	Blocked ROS production, decreased levels of peroxynitrite and abrogated MDSC suppressive activity against antigen-specific CD8+ T cells	>1 μM	[56]

Akt: protein kinase B1;Bad: Bcl-2-associated death promoter; Bcl-2:B-cell lymphoma 2; BCL-xL: B-cell lymphoma extra large; c-FLIP: cellular FLICE inhibitory protein; CHOP: CCAAT/enhancer binding protein homologous protein; COX-2: cyclooxygenase 2; DR:death receptor; ER: endoplasmic reticulum; GSH: glutathione; hTERT: human telomerase reverse transcriptase; JNK: c-Jun NH2-terminal kinase; MDR: multidrug resistant; MDSC: myeloid-derived suppressor cells; mTOR :mammalian target of rapamycin; NAC: N-acetylcysteine; NF-κB: nuclear factor-κB; ROS: reactive oxygen species; STAT3: transcription factor signal transducers and activators of transcription 3; TRAIL :TNF related apoptosis inducing ligand; VEGF: vascular endothelial growth factor; XIAP: X-linked inhibitor of apoptosis protein.

### In vitro studies

#### Targeting nuclear factor kappa B (NF-κB) pathway

The NF-***κ*** B pathway is an important target of the CDDO-me. NF-***κ*** B activates a number of genes that promote inflammation, proliferation, and survival, and because it regulates the inflammatory microenvironment, this pathway can promote tumorigenesis [24,25]. Deeb et al. [26] investigated the response of hormone-sensitive (LNCaP) and hormone-refractory (PC-3 and DU145) human prostate cancer cell lines to CDDO-Me. Their results demonstrated that CDDO-Me could inhibit the growth of prostate cancer cells. Detailed analysis of the antitumor activity of CDDO-Me showed that it induced apoptosis in LNCaP and PC-3 cells through activation of caspases 3, 8 and 9, disruption of mitochondrial integrity, and inhibition of anti-apoptotic B-cell lymphoma 2 (Bcl-2), B-cell lymphoma extra large (Bcl-xL) and X-linked inhibitor of apoptosis protein (XIAP). Furthermore, induction of apoptosis was associated with inhibition of the NF-***κ*** B signaling pathway.

#### Targeting phosphoinositide 3-kinase/ protein kinase B1/ mammalian target of rapamycin (PI3K/Akt/mTOR) pathway

Although the activation of the PI3K/Akt/mTOR pathway may be beneficial in normal retinal epithelial cells or in neurons to enhance survival, this pathway is often overexpressed or activated in cancer cells [27]. Numerous inhibitors of this pathway are being evaluated in clinical trials for the treatment of cancer [28,29]. Deeb et al. [30] determined the antitumor activity and the mechanism of action of CDDO-Me for human prostate cancer cells. CDDO-Me inhibited the growth and induced apoptosis in PC-3 and C4-2 cells at extremely low concentrations. The antitumor activity of CDDO-Me was associated with the inhibition of p-Akt, mTOR and NF-***κ*** B signaling proteins and their downstream targets. Silencing of Akt sensitized the PC-3 cells to CDDO-Me, whereas overexpression of Akt induced resistance to CDDO-Me. Targeted silencing of Akt showed that Akt did not regulate mTOR activation in PC-3 cells, but targeted silencing of mTOR sensitized PC-3 cells to CDDO-Me mediated growth inhibition. These data identified Akt and mTOR as molecular targets of CDDO-Me in prostate cancer cells. In the subsequent study [31], the same group also found that CDDO-Me induced reactive oxygen species (ROS) generation. The inhibition of ROS generation by N-acetylcysteine (NAC) or by overexpression of antioxidant enzymes glutathione peroxidase (GPx) and superoxide dismutase-1 (SOD-1) prevented CDDO-Me-induced apoptosis. NAC also prevented the inhibition of constitutively active Akt, NF-***κ*** B and mTOR by CDDO-Me. The inhibition of PI3K/Akt/mTOR pathway may be a ROS dependent manner. Similar antitumor effect and mechanism of CDDO-Me also reported in pancreatic cancer cells [32,33], colorectal cancer cells [34,35], ovarian cancer cells [36,37], human glioblastoma and neuroblastoma cell lines [38].

#### Targeting janus-activated kinase (JAK) /STAT pathway

STATs are transcription factors known to contribute to cellular transformation, proliferation, survival, invasion, and metastasis, and STAT3 is constitutively activated in many types of cancers. After ligand binding to growth factor receptors, such as the interleukin 6 (IL-6) receptor, a JAK phosphorylates the receptor, allowing recruitment and phosphorylation of a STAT. STATs then dimerize, translocate to the nucleus, and induce transcription of numerous STAT targets, including cyclin D1, myc, and survivin proteins [39,40]. By directly interacting with Cys^1077^ on JAK1, CDDO-Me not only suppresses JAK1 phosphorylation but also inhibits its ability to phosphorylate STAT3 and induce STAT3 dimerization [41]. As both JAK and Src activation could induce STAT3 activation, inhibition of JAK2 or Src is another mechanism for CDDO-Me-mediated inhibition of STAT3 phosphorylation and nuclear translocation. In accordance with this mechanism, CDDO-Me can reverse the multi-drug resistant of ovarian cancer cells [42]. Similar anti-tumor effects and mechanism of CDDO-me are also found in osteosarcomas cancer cells [43].

#### Targeting death receptor (DR) induced extrinsic apoptotic pathway

It is well known that there are two major apoptotic pathways: the intrinsic mitochondria mediated pathway and the extrinsic DR induced pathway [44]. Several studies have demonstrated that CDDO-Me activates both intrinsic and extrinsic apoptotic pathways [45-47]. However, the DR5 related extrinsic apoptotic pathway has been extensively evaluated. Zou et al. showed that CDDO-Me induced DR5 up-regulation, which contributes to CDDO-Me induced apoptosis and enhancement of tumor necrosis factor-related apoptosis-inducing ligand (TRAIL)-induced apoptosis in human non-small cell lung cancer (NSCLC) cells [47]. CDDO-Me depleted intracellular glutathione (GSH), resulting in endoplasmic reticulum stress. Subsequently, it activated c-Jun NH2-terminal kinase (JNK), leading to CCAAT/enhancer binding protein homologous protein (CHOP)-dependent DR5 up-regulation and apoptosis [48]. After that, they further showed that, in addition to DR5 induction, CDDO-Me down-regulated cellular FLICE-inhibitory protein (c-FLIP) levels in human NSCLC cells though JNK-independent ubiquitin/proteasome-mediated degradation of c-FLIP. c-FLIP is a key regulatory protein that inhibits activation of the DR induced extrinsic apoptotic pathway. These studies provide compelling evidence for the involvement of the activation of the DR induced extrinsic apoptotic pathway in CDDO-Me-induced apoptosis in human NSCLC cells [49].

#### Targeting telomere and/or telomerase

Telomeres, the nucleoprotein structures present at the end of chromosomes, play a critical role in maintaining chromosome stability by preventing loss of telomeric repeats, end-to-end fusion and chromosomal rearrangement [50]. Telomeres shorten progressively during normal cell division due to a gradual loss of telomeric DNA sequence (TTAGGG) in each replication cycle [51,52]. When telomere length becomes critically short, it triggers replicative senescence or apoptosis. Maintaining telomere length is the function of telomerase (TERT), a ribonucleoprotein polymerase that adds the hexameric DNA repeats (TTAGGG) to the 3’flanking end of DNA strands in the telomere. Deregulated telomerase activity is associated with promotion of tumorigenesis and neoplastic growth of human cancers [53]. Deeb et al. [54] showed that inhibition of proliferation and induction of apoptosis in pancreatic cancer cells by CDDO-Me involved the inhibition of telomerase activity. CDDO-Me inhibited human TERT gene expression, human TERT protein and human TERT telomerase activity. Further, CDDO-Me also inhibited human TERT regulatory proteins such as c-Myc, specificity protein (Sp)1, NF-κB, p-STAT3 and p-Akt. Thus, targeting the telomere and/or telomerase with CDDO-Me is a promising strategy for the treatment of pancreatic cancer. Subsequently, they investigated the role of ROS in inhibition of telomerase by CDDO-Me. Treatment of MiaPaCa-2 and Panc-1 pancreatic cancer cell lines with CDDO-Me induced the production of hydrogen peroxide and superoxide anions and inhibited the telomerase activity. Pretreatment of cells with NAC, a general purpose antioxidant, or overexpression of GPx or SOD-1 blocked the telomerase inhibitory activity of CDDO-Me. Furthermore, blocking ROS generation also prevented the inhibition of TERT gene expression, protein production and expression of a number of human TERT–regulatory proteins by CDDO-Me. They concluded that inhibition of telomerase activity by CDDO-Me was mediated through a ROS-dependent mechanism [55]. However, the titration effect and direct interaction between CDDO-Me and NAC should be considered when interpreting the results of these studies. Similar anti-tumor effect and mechanism of CDDO-Me also found in prostate cancer cells [23] and ovarian cancer cells [37].

#### Targeting immune cells

Nagaraj et al. [56] reported that CDDO-Me could block immune suppressive function of myeloid-derived suppressor cells (MDSC) and improve immune response of solid tumor. MDSC promote tumor angiogenesis, invasion and suppress T cell mediated immunosuppression of cancer cells [57,58]. They evaluated the effect of the CDDO-Me in MC38 colon carcinoma and Lewis lung carcinoma. They found that CDDO-Me completely abrogated immune suppressive activity of MDSC in vitro. CDDO-Me reduced ROS in MDSC but did not affect their viability or the levels of nitric oxide and arginase. CDDO-Me may favorably improve antitumor immune function, especially when combined with a cancer vaccine.

### In vivo studies

Whether CDDO-Me inhibits tumor growth in vivo was examined in PC-3 prostate cancer xenograft model system [30]. 2 × 10^6^ PC-3 cells in 50 ml of medium mixed with 50 ml of matrigel were injected subcutaneous in the flank of BALB/c nude mice. On day 8, tumor dimensions were measured with a caliper and CDDO-Me treatment started. In the treatment group, mice were gavaged daily with 10 μmol/kg CDDO-Me in 0.1 ml of vehicle consisting of cremaphor EL:DMSO:PBS (1:1:8), 5 days a week for 5 weeks. In the control group, mice were gavaged with vehicle without CDDO-Me. Tumors in the vehicle control group progressively increased in size over a period of 6 weeks and one mouse was terminated on day 39 because of large tumor size. In the CDDO-Me treatment group, after an initial increase in tumor size for about 2 weeks, tumors began to shrink and decreased in size. After 5 weeks of treatment with CDDO-Me, the average tumor size was significantly less than the size on the day treatment was started. On day 50, the experiment was terminated and tumors were harvested and weighed. Tumors of mice treated with CDDO-Me weighed significantly less than those of the untreated mice. These data demonstrated significant antitumor activity of CDDO-Me in vivo.

Jutooru et al. investigated the in vivo anticancer activity of CDDO-Me in an orthotopic model of pancreatic cancer in which L3.6pL cells were injected directly into the pancreas of 8- to 12-week-old male thymic nude mice. Treatment with CDDO-Me (7.5 mg/kg/day) was initiated 7 days after injection of the cells and continued for an additional 28 days. Treatment with CDDO-Me significantly decreased pancreatic tumor volume and weight compared with the vehicle control group. In addition, lysate from tumors treated with the vehicle or CDDO-Me were also analyzed by western blots, and there was a marked decrease in expression of Sp1, Sp3, and Sp4 proteins in tumors from mice treated with CDDO-Me compared with the control group. Moreover, expression of vascular endothelial growth factor (VEGF), cyclin D1, and survivin in tumors from CDDO-Me-treated mice were decreased compared with animals receiving vehicle control [59].

In an immunocompetent mouse model of breast cancer, chemoresistant 4 T1 cells, derived from a spontaneous mammary tumor, were injected back into BALB/c mice to study primary tumors as well as metastases. CDDO-Me at 200 μg per mouse was given intravenous at 2-day intervals. When treatment with CDDO-Me in liposomes was started 1 day after the injection of 4 T1 cells, the drug completely blocked tumor formation and metastasis. Tumor size was also significantly smaller in the mice treated with CDDO-Me than in the control group, even when treatment was delayed until 5 days after inoculation of the aggressive 4 T1 cells. Moreover, the population of mature dendritic cells in BALB/c mice with 4 T1 tumors decreases by two thirds compared with BALB/c mice without tumors, but CDDO-Me helped maintain the number of mature dendritic cells in this model. CDDO-Me not only inhibited the invasion of 4 T1 breast cancer cells into a matrix, but also eliminated metastasis to the lungs when these cells are injected into BALB/c mice [60].

MMTV-neu mice were used to test whether CDDO-Me and the rexinoid LG100268 prevent the formation of ER-negative mammary tumors, or either arrest the growth or cause regression of established tumors. Mice with tumors at least 4 mm in diameter were fed control diet, CDDO-Me (100 mg/kg diet), LG100268 (60 mg/kg diet), or the combination for 4 weeks. Treating established tumors with CDDO-Me arrested the growth of 86% of the tumors, and LG100268 induced tumor regression in 85% of tumors. CDDO-Me and LG100268 targeted different signaling pathways and cell types. CDDO-Me inhibited constitutive STAT3 phosphorylation and the degradation of IKBa in ER-negative breast cancer cells, whereas LG100268 blocked IKBa degradation and the release of IL-6 in RAW264.7 macrophage-like cells, inhibited the ability of endothelial cells to organize into networks, and blocked angiogenesis in vivo [17].

There are undoubtedly other targets and pathways that account for the effects of CDDO-Me on established cancers, but new mechanistic studies will require additional experiments and analysis in relevant animal models.

### **C**linical trials

Most of the experimental evidence favors further development of the triterpenoid CDDO-Me as a potential agent for prevention and treatment of cancer. Clinical trials should be carried out to test the potential efficacy of this drug in solid cancer treatment.

Nagaraj et al. [56] analyzed samples from 19 patients with pancreatic adenocarcinoma that were treated in the phase I clinical trial RTA 402-C-0702. Patients were treated intravenously with gemcitabine (1,000 mg/m^2^) weekly on days 1, 8, and 15. CDDO-Me was administered orally once daily for 21 days. Nine patients received a dose of 150 mg/d; 2 patients, 200 mg/d; 6 patients, 250 mg/d; and 2 patients, 300 mg/d. No toxicity attributed to CDDO-Me was observed. Treatment of pancreatic cancer patients with CDDO-Me did not affect the number of MDSCs in peripheral blood, but significantly improved the immune response.

Hong et al. [61] established a phase I clinical trial which aimed to determine the dose-limiting toxicities (DLT), maximum tolerated dose (MTD), and appropriate dose for phase II studies; to characterize pharmacokinetic and pharmacodynamics parameters; and to assess antitumor activity. They found the DLT to be reversible grade 3 liver transaminase elevation. The MTD was established as 900 mg/d. A complete tumor response occurred in a mantle cell lymphoma patient, and a partial response was observed in an anaplastic thyroid carcinoma patient. NAD(P)H dehydrogenase, quinone 1 (NQO1) mRNA levels increased in peripheral blood mononuclear cells, and NF-***κ*** B and cyclin D1 levels decreased in tumor biopsies. They concluded that CDDO-Me was well tolerated with an MTD of 900 mg/d. For the observed objective tumor responses, other synthetic triterpenoids were suggested for continued development in solid cancer treatment.

## Conclusions

There has been substantial evidence supporting the preventive effect of CDDO-Me in a number of in vivo studies. There is also convincing evidence in laboratory studies and clinical trials in support of the antitumor effects of CDDO-Me in several cancers, with inhibition of tumor cell growth mainly by modulation of pathways that contribute to cell proliferation and apoptosis. Nevertheless, a considerable amount of work remains to be done, such as identification of novel target proteins and intercellular pathways in which they function, and development of selective end points and surrogate biomarkers for evaluating efficacy. Long standing epidemiological studies and well-designed clinical trials are also necessary.

In summary, the in vitro and in vivo data examined in this review strongly suggests that triterpenoid CDDO-Me is a promising candidate for the preventive and treatment of solid cancer.

## Competing interests

The authors declare that they have no competing interests.

## Authors’ contributions

YYW wrote the manuscript. YYW, HZ, and RZ assisted with the revision of English grammar and style. All authors discussed the content and approved the final version of manuscript.
